# Nondestructive Testing for Wheat Quality with Sensor Technology Based on Big Data

**DOI:** 10.1155/2020/8851509

**Published:** 2020-11-20

**Authors:** Yan-Ge Tian, Zheng-Nan Zhang, Shuang-Qi Tian

**Affiliations:** ^1^Henan University of Technology, High-tech Development Zone, Zhengzhou 450001, China; ^2^Zhengzhou Electronic ＆ Information Engineering School, Zhengzhou 450007, China

## Abstract

Nondestructive testing with sensor technology is one of the fastest growing and most promising wheat quality information analysis technologies. Nondestructive testing with sensor technology benefits from the latest achievement of many disciplines such as computer, optics, mathematics, chemistry, and chemometrics. It has the advantages of simplicity, speed, low cost, no pollution, and no contact. It is widely used in wheat quality analysis and testing research. This article summarizes nondestructive testing with sensor technology for wheat quality, including the mechanical model, hyperspectral technology, Raman spectroscopy, and near-infrared techniques for wheat mechanical properties, storage properties, and physical and chemical properties (such as moisture, ash, protein, and starch) in the past decade. Based on the current research progress, big data technology needs a lot of research in spectral data mining, modeling algorithm optimization, model robustness, etc. to provide more data support and method reference for the research and application of wheat quality.

## 1. Introduction

Wheat is one of the staple foods of humans, and wheat flour is the main processed product of wheat, which occupies a large proportion in people's diet structure [1, 2]. It can be made into bread, noodles, Chinese steamed bread, dumplings, and other traditional foods. The quality of wheat will directly affect the quality of downstream wheat products, but also is related to the physical and mental health and economic interests of consumers [3]. With the improvement of people's lifestyles and the continuous improvement of quality of life, higher requirements have been placed on the quality and type of food, especially the demand for various foods made from wheat. To meet the needs of the market consumption structure upgrade, the promotion and application of high-quality special wheat has become an important part of modern agricultural research [4]. In the process of wheat quality analysis, simple and effective detection methods can be used to timely understand the quality of wheat and provide an effective value for the breeding and eating quality of wheat varieties.

Nondestructive testing with sensor technology has the advantages of easy operation, high efficiency and stability, good repeatability, and no damage to samples. It is widely used in the measurement and analysis of wheat quality [5]. Nondestructive testing with sensor technology is a high-sensitivity detection device that uses radiation, infrared, electromagnetic, and other principles combined with instruments to detect defects and chemical and physical parameters of wheat without damaging or affecting the performance of the tested object [6]. This article summarizes the mechanical model, hyperspectral technology, Raman spectroscopy, and near-infrared techniques in wheat mechanical properties (hardness, compressive strength, and shear capacity), storage properties (enzyme activity and germination rate), and physical and chemical properties (such as moisture, ash, protein, and starch) in the past decades.

## 2. Application of Nondestructive Testing with Sensor Technology in Wheat Quality

Wheat is the main food for people to maintain life. It is of great practical significance to analyze the storage and nutritional characteristics of wheat and provide a scientific basis for people's reasonable diet [7]. For wheat quality analysis, different indicators require different analysis methods [8]. Mechanical analysis and spectroscopy analysis are the fast and nondestructive testing methods, which have become the main methods of agricultural product quality analysis [9, 10].

### 2.1. Application of Mechanical Model Sensor Technology in Wheat Quality

The mechanical properties of wheat refer to various external loads (hardness, compressive strength, shear capacity, etc.) under different environments. The force and deformation characteristics of wheat piles under complex load conditions are an important reference for the design of grain storage buildings and an important part of safe storage of wheat and ensuring wheat quality. As shown in Table 1, the results of part of the wheat mechanical property test are displayed, which can quickly determine the stress of wheat.

The triaxial test of wheat is a relatively complete experimental method to determine the mechanical properties of wheat in the current mechanical research and engineering practice. The triaxial test device is mostly a strain-controlled triaxial instrument, which uses a resistance strain sensor to make a local strain gauge. The main parameters of wheat grain piles are elastic modulus, dilatancy angle, and internal friction angle. The elastic modulus of the grain pile reflects the relationship between stress and strain within the elastic range of the grain pile, and it is also an important mechanical characteristic of the grain pile [11]. The internal friction angle is a characterization of the interaction between grains in the grain pile, and its value has a great influence on the state of wheat grain storage. The internal friction angle is a parameter of grain strength widely used in the specification. In terms of the deformation of the wheat pile, the overall volume of the wheat pile will become larger when it is sheared [12]. The dilatancy angle is used to describe the characteristics of the pile volume change, which is a quantitative expression of the dilatancy characteristics [13]. Taking wheat as the research object, the range of internal friction angle of the wheat pile was determined by using the direct shear test device with a vertical pressure range of 2.8–48 kPa and different displacement velocities. The test shows that the internal friction angle decreases with the increase of the normal pressure, and the shear resistance of the material decreases with the increase of the water content and the increase of the loading rate. As the displacement increases, the deformation is limited to the shear area. When the shear stress of the specimen reaches its maximum value, the thickness of the area where the specimen undergoes shear stabilizes [14, 15].

The conventional triaxial test can be used to test the mechanical properties of the grain. The triaxial test can not only study the stress and strain performance but also be an important method for other mechanical performance testing. At the same time, it overcomes the shortcomings of the fixed failure surface in the direct shear test, and the test results are more in line with the regulations.

**Figure 1:**
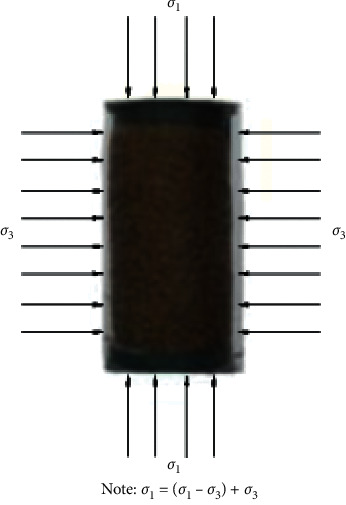
Schematic diagram of the force in the triaxial test of wheat.

The triaxial test can simulate the local stress environment of the wheat grains in the granary and the mechanical properties of the wheat during loading and unloading.  Figure 1 shows a schematic diagram of the force in the triaxial test of wheat. At the same time, the stress-strain model of the wheat can be obtained, and the elastic modulus, dilatancy, and internal friction angle of the wheat can be obtained. These parameters can support granary design and numerical simulation analysis. The experiment was carried out by using a triaxial instrument. The normal stress was between 4 and 40 kPa, and the three samples prepared under different initial densities were tested for 5 times. The results showed that the internal friction angle decreased with the increase of the volume density. In addition, a strength model was established based on 36 sets of triaxial tests under different combinations of wheat hardness, moisture content, and density, which explained the curvature change of the molar strength envelope [16, 17]. In the triaxial test, different confining pressures can be considered to simulate the mechanical properties of wheat in different storage locations, and the mechanical properties of wheat in different initial states can be obtained by considering different void ratios. Zhang considered four different confining pressures, and Bock conducted a triaxial test of wheat under different confining pressures, moisture content, and initial density and obtained the strength parameters of wheat in different initial states [18, 19]. Zeng used an improved triaxial apparatus for grain under 3 different confining pressures (50, 100, and 200 kPa), while considering the initial void ratio of wheat and the shear rate of the experiment, 21 triaxial compression tests were carried out. It is concluded that the shear speed has an effect on the shear strength, internal friction angle, and cohesion. The angle of internal friction increases with the decrease of porosity. In addition, the dilatancy angle decreases with the increase of confining pressure [20]. The triaxial test can also test the mechanical properties of wheat flour. When the confining pressure is 100, 200, and 300 kPa, the cohesion of wheat flour is 53 kPa. The result shows that the internal friction angle is 29.50°, and the cohesion of concentrated wheat flour is 47 kPa. The internal friction angle is 21.05° [21, 22].

**Figure 2:**
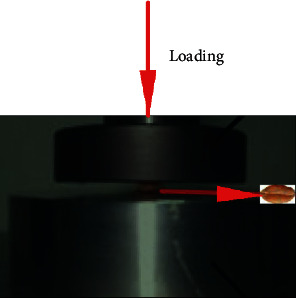
Schematic diagram of the force of the wheat uniaxial test.

In the process of wheat processing, storage, and transportation, the crushing of grains has always been a major problem. The crushing of grains not only causes a huge waste of grain but also brings a series of problems to its transportation, storage, and production.  Figure 2 shows a schematic diagram of the force in the uniaxial test of wheat. The uniaxial test can measure the elastic modulus of a single wheat grain and the external load that the wheat bears when it is broken. Molenda considers 5 different water contents and concludes that the elastic modulus and Poisson's ratio of wheat decrease with the increase of water content [23, 24]. Voicu selected three kinds of wheat seeds, performed a uniaxial compression test on 50 wheat seeds of each type of seeds, and recorded the force-deformation curve of the wheat during the test. The average force-deformation curve was drawn, respectively, and the results showed that at the same strain value, the three wheat varieties showed similar mechanical behavior in the uniaxial compression test, but the breaking force value and average breaking energy were different [25]. The uniaxial compression test under a small strain (strain of 3%) is used to evaluate the viscoelastic properties of a single wheat grain, which shows that the elasticity theory can be used to explain the viscoelasticity of wheat grains and their relationship with physical and chemical properties. The results show that viscoelasticity is significantly negatively correlated with thousand-grain weight, thousand-grain weight, and grain thickness. The uniaxial compression test under small-strain conditions can be used to evaluate the viscoelastic properties of wheat grains of different grades and varieties [26]. In addition, the force-deformation curve of the wheat uniaxial compression test has at least two inflection points. The first inflection point is the mechanical properties of the bran layer, and the second inflection point is related to the endosperm boundary near the aleurone layer [27]. When wheat goes through the process of wetting and drying, the heat and mass of wheat particles will change. This process will cause the change of stress inside the grains, which will lead to the endosperm rupture, and the grains will produce cracks. These cracks will cause changes in the mechanical properties of wheat. At the same time, it is found that there is a significant negative correlation between the mechanical properties of wheat particles and the wetting time. Similarly, a highly significant correlation was found between the mechanical properties and the number of internal cracks [28]. However, the mechanical model with sensor technology could not reflect physical and chemical properties in wheat quality.

### 2.2. Application of Near-Infrared Spectroscopy and Hyperspectroscopy Sensor Technology in Wheat Quality

Hyperspectral sensing technology has the characteristics of map integration, which can detect the fine spectral characteristics of elements that are lacking in wide-band imaging remote sensing, which is essential for fine quality identification and parameter inversion. Near-infrared light is an electromagnetic wave with a wavelength between the visible light region and the mid-infrared region. The wavelength range is between 780 and 2526 nm. It is the first invisible light discovered by people. Near-infrared spectroscopy technology is to use the material information contained in the near-infrared spectral analysis, which has many applications in wheat quality.  Table 2 shows the test data of wheat using near-infrared spectroscopy analysis technology and analyzes the main application aspects of near-infrared analysis technology.

For example, in terms of variety identification, using near-infrared diffuse reflectance technology, with hard white wheat, hard red spring, hard red winter, soft red winter, and soft white wheat planted in 1987, 1988, 1989, and 1990 as the research objects, 100 samples of wheat grading were systematically studied every year, and the correct grading of each variety of wheat was obtained [29]. Marcott collected 4,096 images and analyzed that the near-infrared spectroscopy analysis technology can clearly distinguish the single-cell layer near the outer shell of the wheat grain and the primary roots in the embryo [30]. Melchor conducted a study on the feasibility of a near-infrared reflectance spectrometer as a granulation sensor for six kinds of winter wheat. At the same time, two sets of 35 wheat samples were tested, and a near-infrared reflectance-granulation model was developed and verified. These models were then compared with the previously developed granulation model, which predicted 34 out of 35 experiments and proved the feasibility of the developed model [31]. It can also be used to classify heat-damaged and nondamaged wheat grains [32].

Wheat scab is one of the main diseases of wheat, which occurs widely in the world. Near-infrared spectroscopy can also be used to detect the damage degree of wheat scab, and the content of deoxynivalenol and ergosterol in wheat grain can be determined. The accuracy level obtained shows the application potential of near-infrared spectroscopy in the detection of wheat scab [33, 34]. Wheat hardness refers to the resistance encountered when the grain is broken. It is determined by the binding strength between the protein matrix and starch in the wheat endosperm cells. It is one of the important phenotypic characteristics of wheat. It directly affects the milling quality of wheat and determines the quality of wheat. It is one of the important indicators of end use, quality, and international trade of wheat [35, 36]. The single kernel characterization system (SKCS) was used to collect wheat single-grain reflectance spectra, and at the same time, the hardness was analyzed by partial least-square regression. The results confirmed that near-infrared reflectance spectroscopy technology quickly and nondestructively was used to measure the hardness of the bulk wheat grains [37]. The use of near-infrared reflectance technology can also study the heritability of grain hardness. Four separate populations were cultivated from three cross combinations, and the heritability estimate of wheat hardness was significant [38]. The prediction of wheat grain hardness is also an important aspect. Zhang proposed a band selection method combining the ant colony algorithm and support vector regression. This method can be well applied to the prediction of wheat grain hardness [39].

In the daily diet, wheat flour is an indispensable and important component, and its quality is related to people's health. The protein in wheat affects the quality of wheat flour. Scholz selects 6 different wheat backgrounds, each with a certain amount of flour or grains, and uses near-infrared reflectance spectroscopy and near-infrared transmission spectroscopy to detect the difference in the quantity and size distribution of wheat polymer protein possibility [40]. Baslar conducted experiments on 120 wheat samples from different regions of Turkey to determine the wet and dry gluten content and Zeleny sedimentation of the wheat [41]. Mao collected 140 high-quality samples from more than a dozen major wheat-producing areas in China, using the near-infrared reflectance spectroscopy and radial basis function (RBF) neural network algorithm to more conveniently determine the protein content of wheat [42]. In terms of quality prediction, combining the bread wheat flour near-infrared spectroscopy data set (391 samples in two regions) and combining different regression methods, the benefits of nonlinear modeling and the advantages of multitarget prediction are obtained [43]. At the same time, there is also a portable near-infrared spectroscopy analysis system, combined with appropriate chemometric methods, to achieve the quantitative determination of fatty acid value during wheat flour storage [44].

Hyperspectral imaging is a powerful technique that combines the advantages of near-infrared spectroscopy and imaging technology, providing information about the chemical properties of objects and their spatial distribution.  Table 3 shows the application of hyperspectral technology in monitoring wheat quality.

Diseases and insect pests have always been an important aspect of harming wheat quality. Singh selected 300 healthy wheat grains and 300 wheat grains destroyed by insects for hyperspectral imaging. The hyperspectral image was collected from 1000 to 1600 nm wheat grains. 85–100% of healthy wheat and wheat with pests and diseases are correctly classified [45]. In addition, when the wavelength range is 700–1100 nm, hyperspectral scanning and color imaging of healthy wheat and wheat kernels damaged by insect feeding were performed. A total of 230 features were extracted from the color image, and the first 10 features of the 230 color image features combined with the hyperspectral image features were used to correctly identify 96.4% of healthy wheat and 91.0–100.0% of insect-infested wheat grains [46].

For other wheat diseases, such as smut, 48 grains were randomly selected from wheat with smut and normal wheat, and the wheat grains were studied using hyperspectral imaging technology. Through the rough inspection of the wavelength image, a fluorescence wavelength (531 nm) is selected for image processing and classification analysis. The results show that only using this wavelength, the classification accuracy can be as high as 95% [47]. In addition, in the classification method of different types of wheat head blight, the reflectance spectra of these three types of wheat grains were obtained in the near-infrared band (1000–1700 nm) by using a laboratory device equipped with an HSI system. Partial least squares discriminant analysis (PLS-DA) was used to classify the three types of wheat. The results showed that the experiment not only considered the entire research wavelength range but also selected 4 best wavelengths (1104, 1384, 1454, and 1650 nm) among 121 wavelengths, and obtained good classification results [48]. Singh comprehensively considered storage wheat diseases and selected 20 kg of wheat for the experiment. The wheat grains infected by the stored fungi species *Penicillium* and *Aspergillus niger* were scanned, and the shortwave near-infrared in the range of 700–1100 nm was selected. Using multivariate image analysis methods to reduce the dimensionality of hyperspectral data and using principal component analysis to select effective wavelengths, studies have shown that the 870 nm wavelength is mainly used in classification and feature extraction [49].

Like near-infrared spectroscopy, hyperspectroscopy is also effective in detecting wheat protein content. On a single-grain basis, 79 sets of wheat flour are selected to determine the distribution of whole wheat protein content, and HSI is used to predict this distribution for complete prediction. The protein content of a single grain could be accurately predicted [50]. Hyperspectral analysis technology has the ability to predict wheat protein content, germination damage, and *α*-amylase activity based on near-infrared spectroscopy. This analysis technology provides classification of grains based on characteristics such as variety, geographic origin, and core hardness [51]. In addition, the performance of hyperspectral imaging instrument and classical near-infrared instrument in predicting chemical composition was compared. Taking the determination of wheat flour protein content as an example, two classical near-infrared instruments and a near-infrared hyperspectral line scanning camera were used to collect spectra in a single sealed chamber. In the latter, in order to study the possibility of accelerating the measurement process, it was obtained in open cells. The calibration model of the full-wavelength range of each instrument and the common range (1120–2424 nm) between the instruments was established by using the partial least squares method. When all instruments and the same sample set use a common wavelength range, the hyperspectral line scanning system works as the classic near-infrared spectrometer, but the time required for the hyperspectral line scanning system to analyze samples is reduced by at least half [52]. However, NIR spectroscopy technology is difficult to model.

### 2.3. Application of Raman Spectroscopy Sensor Technology in Wheat Quality

Raman spectroscopy is a new type of rapid detection technology that can enhance the sensitive detection of the molecular information of the compound through the sensor, so as to clearly analyze its characteristic chemical structure. It has simple sample preprocessing, fast detection speed, high sensitivity, and rich spectral information. Raman spectroscopy has great application value in the field of wheat quality detection.  Table 4 shows the application of Raman spectroscopy in monitoring wheat quality. Raman spectroscopy is a scattering spectrum, which is used to obtain information such as molecular vibration and rotation by analyzing the scattering spectrum with a different frequency from the incident light, which can be used for the study of molecular structure [53]. In the study of wheat embryo and endosperm, Raman spectroscopy revealed that in the tolerant genotype, protein mainly accumulates in the embryo, and the whole grain protein content in the tolerant genotype and the sensitive genotype is similar [54]. Confocal Raman spectroscopy is a comprehensive application of Raman spectroscopy and microscopy. A total of 100 Raman spectra are recorded at different points of 10 different endosperm cell walls in the same sample group to study the protein content of wheat grain starch endosperm. The results showed that the role of the endosperm cell wall in grain structure and cohesion was the molecular basis of grain hardness. It can also identify the effectiveness of specific molecular factors that cause grain agglomeration and participate in the fracture pattern produced during the grinding process [55]. At the same time, confocal Raman spectroscopy can be used to locate the particle components. Two methods are used to process and image 12 samples of each grain tested. The results show the detailed distribution of substances on the aleurone layer cell wall [56]. In the comprehensive application of microscopy and Raman spectroscopy, secondary field emission scanning electron microscopy, atomic force microscopy, and Raman spectroscopy can be used in combination with X-ray photoelectron spectroscopy to analyze near-isogenic soft and hard wheat grains and their milling were studied. It provides a new perspective to further understand the difference between soft and durum wheat grains [57].

In terms of nondestructive screening and classification, Raman spectroscopy can be used to quickly and nondestructively screen DON-contaminated wheat and barley flour [58]. A near-infrared spectrometer was used to collect the sample spectrum of wheat seeds, and then, a Raman spectrometer was used to collect the sample spectrum. All samples are randomly divided into a calibration sample set, which contains 284 seeds (about 35 seeds per row), and a verification sample set that contains the remaining 92 seeds. According to the raw spectra that have been properly preprocessed, these samples are classified using a combination of discriminant analysis and principal component analysis. The mutant wheat lines were classified. The combination of Raman spectroscopy and chemometrics is superior to near-infrared spectroscopy in the classification of wheat strains [59].

Raman spectroscopy was used to detect wheat tissues, and the mature grains of 3 common wheat varieties and 1 durum wheat were manually dissected. Using Raman spectroscopy to collect vibration spectrum characteristics on the film combined with statistical analysis, the specific spectral characteristics of each peripheral tissue of wheat were identified successfully [60]. However, the fluorescence phenomenon interferes with the analysis of Raman spectroscopy, and the problem of nonlinearity of the curve often occurs.

## 3. Conclusions

Nondestructive testing is a technology that uses radiation, infrared, electromagnetic, and other principles combined with instruments to detect defects, and chemical and physical parameters of wheat without damaging or affecting the performance of the tested object. The triaxial test can consider the initial conditions of the test, simulate the stress path including loading and unloading, and provide the grain stress-strain model. Big data analysis of the wheat triaxial test can provide technical support for wheat storage. Big data analysis of the wheat uniaxial test can understand the mechanical properties of wheat grains, thereby reducing the compression and impact of wheat grains and improving wheat quality. Near-infrared spectroscopy is also widely used in wheat quality. Near-infrared spectroscopy analysis technology can detect the hazard degree of wheat grain scab, and nondestructively measure the potential of wheat grain hardness, the distribution of the quantity, and size of wheat polymer protein. Hyperspectral technology combines the advantages of near-infrared spectroscopy and imaging technology, and is outstanding in the detection of wheat diseases and insect pests and protein content. Raman spectroscopy is also mainly used in these areas. Big data can reflect the application of spectroscopy technology in wheat quality detection and screening, and provide technical support for wheat quality research. Nondestructive testing technology can also be applied to other grains, fruits and vegetables, and hazardous factor testing in the future.

## Figures and Tables

**Table 1:** Mechanical test of wheat.

No.	Type	Data	Conclusion	References
1	Triaxial test	15	The internal friction angle decreases with the increase of bulk density.	[16]
2	Triaxial test	36	The curvature variation of the molar strength envelope is explained.	[17]
3	Triaxial test	15	The estimated fourteen parameters were used in the constitutive equation to calculate stress-strain relationships for wheat en masse in axial compression and isotropic compression loading conditions.	[18]
4	Triaxial test	21	The shear rate has influence on shear strength, internal friction angle, and cohesion.	[20]
5	True triaxial tests	30	The mechanical properties of wheat flour and the change of curve were predicted by constitutive relation.	[21]
6	Uniaxial test	150	The three wheat varieties showed similar mechanical behaviors in the uniaxial compression test, but the breaking force value and average breaking energy were different.	[25]
7	Uniaxial test	16	The uniaxial compression test under small-strain conditions can be used to evaluate the viscoelastic properties of wheat grains of different grades and varieties.	[26]
8	Uniaxial test	18	The force-deformation curve has at least two inflection points, the first inflection point is the mechanical properties of the bran layer, and the second inflection point is related to the endosperm boundary near the aleurone layer.	[27]

**Table 2:** Near-infrared spectroscopy test of wheat.

No.	Data	Conclusion	References
1	400	The correct grading of various varieties of wheat.	[29]
2	4096	Distinguishing the single cell layer near the outer shell of the wheat kernel and the primary roots in the germ.	[30]
3	70	A near-infrared reflection-granulation model was developed and verified.	[31]
4	60	Visible light and near-infrared reflectance spectroscopy technology are used to quickly and nondestructively measure the hardness of bulk wheat grains.	[37]
5	86	A band selection method combining ant colony algorithm and support vector regression is proposed to predict wheat grain hardness.	[39]
6	192	Possibility to detect differences in the quantity and size distribution of wheat polymer protein.	[40]
7	120	The wet and dry gluten content and Zeleny sedimentation of wheat were measured.	[41]
8	140	Near-infrared reflectance spectroscopy and radial basis function (RBF) neural network algorithm can be more convenient for the determination of wheat protein content.	[42]
9	391	Combined with different regression methods, the advantages of nonlinear modeling and multiobjective prediction are obtained.	[43]

**Table 3:** Hyperspectral test of wheat.

No.	Data	Conclusion	References
1	600	The correct classification of 85–100% of healthy wheat and wheat with pests and diseases is carried out.	[45]
2	230	96.4% of healthy wheat and 91.0–100.0% of pest-infested wheat kernels are identified.	[46]
3	48	A fluorescence wavelength of 531 nm was chosen for image processing and classification analysis, and the classification accuracy can be as high as 95%.	[47]
4	121	In the classification methods of different types of wheat head blight, only 4 optimal wavelengths (1104, 1384, 1454, and 1650 nm) were selected among 121 wavelengths, and good classification results were obtained.	[48]
5	79	Determining the distribution of whole wheat protein content.	[50]
6	190	Taking the determination of protein content of about 190 wheat flour as an example, the hyperspectral line scan system works like a classic near-infrared spectrometer, but the time required to analyze the sample is reduced by at least half.	[52]

**Table 4:** Raman spectroscopy test of wheat.

No.	Data	Conclusion	References
1	180	In the tolerance genotype, protein mainly accumulates in the embryo, and the whole grain protein content in the tolerance genotype and the sensitive genotype is similar.	[54]
2	100	The role of endosperm cell wall in grain structure and cohesion is the molecular basis of grain hardness.	[55]
3	1000	The near-isogenic soft and hard wheat grains and their milled flour were studied.	[57]
4	768	Fast and nondestructive screening of DON-contaminated wheat and barley flour.	[58]
5	376	A combination of discriminant analysis and principal component analysis is used to classify these samples. The mutant wheat lines were classified.	[59]
6	25	The combination of FTIR spectroscopy and statistical analysis successfully identified the specific spectral characteristics of each peripheral tissue of wheat.	[60]

## Data Availability

The data used to support the results of this study are included within the article.
